# Search for *Equus caballus* papillomavirus type 2 in tissues of asymptomatic horses in southern brazil

**DOI:** 10.1007/s42770-026-01930-y

**Published:** 2026-04-22

**Authors:** Eduarda Maria Trentin Santi, Joana Braga Pecego, Maria Fernanda Abel Schirmer, Thainã Piccolo Vargas, Valentina Berté Marcus, Indianara de Vargas, Glaucia Denise Kommers, Mariana Cocco, Nathalia de Freitas Michelon, Eduardo Furtado Flores, Mariana Martins Flores

**Affiliations:** 1 Programa de Pós-Graduação em Medicina Veterinária, Universidade Federal de Santa Maria, Santa Maria, RS Brasil; 2 Curso de Graduação em Medicina Veterinária, Universidade Federal de Santa Maria, Santa Maria, RS Brasil

**Keywords:** EcPV-2, Carrier, Subclinical, Brazil, South America, Equine

## Abstract

*Equus caballus* papillomavirus type 2 (EcPV-2) has been recently associated with the development of genital squamous cell carcinomas in horses. Nevertheless, its circulation among healthy, asymptomatic horses remains poorly understood. This study aimed to investigate the presence of EcPV-2 DNA in asymptomatic horses submitted to necropsy in southern Brazil. A total of 54 horses without clinical or histological evidence of genital lesions were investigated: 40 necropsied animals and penile mucosa specimens collected from 14 horses at a local abattoir. Tissue samples were subjected to histological analysis and polymerase chain reaction targeting a 306 bp fragment of the EcPV-2 L1 gene. EcPV-2 DNA was detected in one animal (1.85%). The sample was obtained from a necropsied male horse that died due to acute intestinal disease. Sequencing of the amplicon obtained in the PCR confirmed the identity of the viral fragment, and phylogenetic analysis clustered the sequence with known EcPV-2 isolates. No histological lesions were observed in any of the tissues examined from the animal containing amplifiable EcPV-2 DNA. This is the first report of EcPV-2 DNA detection in the genital mucosa of an asymptomatic horse in Brazil, supporting the possibility of subclinical infection. These findings provide initial evidence that EcPV-2 may occur in clinically normal horses in Brazil and highlight the need for further studies to determine the prevalence of subclinical infection and the potential role of asymptomatic carriers in viral transmission.

**Supplementary Information:**

The online version contains supplementary material available at 10.1007/s42770-026-01930-y.

## Introduction

The Papillomaviridae family comprises DNA viruses widely distributed among humans and domestic animals, some of which are known for their oncogenic potential [1]. To date, at least ten equine papillomaviruses (EcPVs) have been described, several of which have been associated with proliferative lesions in horses. These include cutaneous papillomatosis (EcPV-1), genital papillomas and squamous cell carcinoma (SCC) (EcPV-2), aural plaques (EcPV-3, -4, -5, and − 6), and vulvar or inguinal plaques (EcPV-4) [2]. EcPV-2 was the first equine papillomavirus associated with malignant epithelial neoplasia (SCC) in horses [3]. More recently, EcPV-7 has also been identified in equine penile SCC [4].

Squamous cell carcinoma (SCC) is a malignant neoplasm of the squamous epithelium, typically arising in the skin or mucous membranes, and is one of the most common malignant tumors affecting the penis and prepuce of horses [5]. Just over a decade ago, EcPV-2 genital infection was associated with genital papillomas and SCC in horses, similar to the role of human papillomavirus (HPV) in cervical cancer [3, 6, 7]. Since then, several studies have explored the presence of EcPV-2 in equine genital papillomas and SCC [8–13]. However, as EcPV-2 was only identified in 2009, its epidemiology, transmission pathways, and pathogenesis remain poorly understood.

Studies investigating the presence of EcPV-2 in asymptomatic horses have been conducted in New Zealand [13], Belgium [11], Austria [10], Switzerland [14], Canada [15], and Italy [16]. In Brazil, studies confirming the detection of EcPV-2 in equine genital SCC are still limited [17], and investigations in asymptomatic horses are lacking. Although subclinical infection was once considered rare [13], recent findings have reported antibodies against EcPV-2 in 36% of horses and detection of EcPV-2 DNA by PCR 29–30% of tissue samples from asymptomatic horses [15, 16]. These findings suggest that clinically normal horses may harbor the virus and potentially contribute to its transmission, although further evidence is still needed.

A better understanding of transmission dynamics, risk factors, pathogenesis, and geographic distribution of EcPV-2 is essential for the development of effective prevention and control strategies. A parallel can be drawn with decades of research on HPV, which enabled the development of vaccines that have significantly reduced cervical cancer incidence in vaccinated populations [18]. In this context, the present study investigated the detection of EcPV-2 DNA in tissues from asymptomatic horses submitted to necropsy in southern Brazil.

## Materials and methods

A total of 54 horses were included in this study. Samples were obtained from 40 horses necropsied at the Veterinary Pathology Laboratory (LPV) of the Federal University of Santa Maria (UFSM) between 2022 and 2025, and from 14 additional horses sampled at a local abattoir. The inclusion criteria were absence of clinical signs and absence of gross or microscopic genital lesions.

For the necropsied horses (*n* = 40), epithelial tissues collected from each animal included penile or vulvar mucosa, anal mucosa, lip, lower eyelid, and stomach. Two samples of each tissue (approximately 1 × 1 cm) were collected: one was fixed in 10% buffered formalin and processed for paraffin embedding, and the other was stored frozen at − 20 °C for molecular analysis. Animal information (sex, age, breed) and cause of death or reason for euthanasia was recorded for each animal. For molecular investigation, frozen epithelial tissues from each necropsied horse were pooled prior to DNA extraction, generating a single DNA sample per animal. This approach was adopted as a screening strategy to increase the likelihood of detecting EcPV-2 DNA in asymptomatic horses rather than to assess tissue-specific viral distribution.

Additionally, penile and preputial mucosal tissues were collected from 14 horses at a local abattoir between 2022 and 2023 and submitted to LPV-UFSM for inclusion in this study. As with the necropsied animals, two penile mucosal tissue samples (approximately 1 × 1 cm each) were collected from each specimen, one fixed in 10% buffered formalin and the other frozen at − 20 °C for molecular analysis.

Frozen samples (*n* = 54), each representing one individual horse, were subjected to molecular investigation for papillomavirus DNA. DNA extraction was performed using DNAzol reagent (Invitrogen, USA) according to the manufacturer’s instructions, with minor modifications. Briefly, 1 mL of DNAzol was added to 25–50 mg of tissue previously fragmented with a scalpel blade to ensure a homogeneous mass. Cells were lysed by pipetting and vortexed for 1 min. Samples were centrifuged at 11,000 RPM for 10 min at room temperature, and the supernatant was transferred to a new tube. Absolute ethanol (100%) was added at a volume of 500 µL per 1 mL of DNAzol used, followed by gentle inversion and incubation at − 20 °C for at least 1 h. DNA was precipitated by centrifugation at 11,000 RPM for 8 min; the supernatant was discarded, and the pellet was washed twice with 1 mL of 75% ethanol. After final centrifugation, the ethanol was discarded, the pellet air-dried and resuspended in 50 µL of TE buffer. DNA quality was monitored in a spectrophotometer (260/280 OD ratio).

Extracted DNA was subjected to PCR specific for EcPV-2. Primers were designed to amplify a 306 bp fragment of the L1 gene based on EcPV-2 isolates (HM461973.1 and EU503122.1), as described by Greenwood et al. (2020). The primer sequences were: EcPV-2For (5’-TCCTCCACCAATTTTAAAACCTAT-3’) and EcPV-2Rev (5’-ATCCAAGTCAAGGGAAAG-3’). PCR reactions were performed in a final volume of 25 µL containing 2 µL of extracted DNA, 0.5 µM of each primer, 2.5 mM MgCl₂, 10 mM dNTPs, 1× buffer, and 1 µL of recombinant Taq DNA polymerase (Thermo Fisher Scientific, USA). Thermocycling conditions included initial denaturation at 94 °C for 3 min, followed by 35 cycles of 94 °C for 30 s, 58.4 °C for 30 s, and 72 °C for 1 min. A final extension was performed at 72 °C for 10 min, followed by storage at 4 °C. Amplified products were visualized by electrophoresis on 2% agarose gels stained with Gel Red^®^ (Biotium, USA) under UV light. DNA extracted from a genital SCC containing EcPV-2 DNA served as a positive control, and ultrapure water as a negative control.

The amplicons obtained were subjected to nucleotide sequencing. PCR products were purified using the PureLink PCR Purification Kit (Invitrogen), according to the manufacturer’s instructions. PCR products were sequenced using forward and reverse primers. Chromatograms were inspected and assembled into a consensus sequence. Sequence identity was assessed using BLAST against sequences available in GenBank. Representative sequences of Equine papillomavirus type 2 were retrieved from GenBank for phylogenetic analysis. Sequences were aligned using the MUSCLE algorithm implemented in Geneious. The alignment was trimmed to the region corresponding to the amplified fragment (~ 200 bp). A phylogenetic tree was constructed using the neighbor-joining method with 1000 bootstrap replicates, and the outlier group corresponded to Equine papillomavirus type 1 (ECPV1). Access to genetic heritage was registered in SisGen (protocol AE43670) as required by Brazilian Law No. 13,123/2015.

All paraffin-embedded tissue samples from horses with detectable EcPV-2 DNA were sectioned at 3 μm and stained with hematoxylin and eosin. Histological sections were examined for the presence of microscopic lesions.

## Results

The studied population comprised of 54 horses. The 40 necropsied horses ranged in age from 14 days to 25 years and included 19 females and 21 males. Breeds included Criollo, Thoroughbred, Brazilian Sport Horse, Mixed Breed, Quarter Horse, and Lusitano (Supplementary Table 1). Information regarding the 14 horses from the local abattoir were not available.

Of the 54 horses analyzed by EcPV-2–specific PCR, only one (1.85%) yielded amplification of the 306-bp fragment of the L1 gene (Table 1). The sample containing amplifiable EcPV-2 DNA was obtained from a male horse necropsied at LPV-UFSM and corresponded to a pooled DNA sample derived from multiple epithelial tissues, including penile mucosa, anal mucosa, lip, lower eyelid, and stomach. The horse died spontaneously due to severe small intestinal intussusception and consequent endotoxemia. DNA extracted from this sample produced a single distinct band on a 2% agarose gel under UV light, with an intensity comparable to that of the positive control. No amplification was observed in the remaining samples or in the negative control, confirming the specificity of the reaction. Histological examination of the penile mucosa, anal mucosa, lips, lower eyelid, and stomach of the horse in which EcPV-2 DNA was detected was unremarkable. Nucleotide sequencing of the obtained amplicon revealed 99% identity with *Equus caballus* papillomavirus type 2 DNA E200929 (Genbank ID: LC12601.1), confirming the identity of the amplicon/amplification. A phylogenetic tree was generated to demonstrate genetic relationship of the identified sequence with other EcPV-2 sequences (supplementary Fig. 1).

**Table 1:** Detection of *Equus caballus* papillomavirus type 2 in tissues of asymptomatic horses: animal source, tissues collected, pooling strategy, and EcPV-2 PCR results

Sample Source	Number of animals	Tissues collected per animal	Pooling strategy for PCR	Number of animals with amplifiable EcPV-2 DNA
Necropsied horses	40	Penile or vulvar mucosa, anal mucosa, lip, lower eyelid, stomach	All epithelial tissues from the same animal were pooled prior to DNA extraction	1
Abattoir horses	14	Penile mucosa	No pooling (single tissue sample per animal)	0

## Discussion

*Equus caballus* papillomavirus type 2 (EcPV-2) was first identified in 2009, primarily in genital papillomas and squamous cell carcinomas (SCCs) of male horses [3, 6]. Most studies on EcPV-2 have focused on its detection in genital papillomas and SCCs, where the virus is frequently identified [7, 8, 19, 20]. Investigations in asymptomatic horses remain limited, and no studies have been conducted in Brazil [15, 16]. This gap limits our understanding of subclinical viral circulation, transmission pathways, and the potential role of asymptomatic carriers. One of the first studies on the topic reported that EcPV-2 sequences were more frequently detected in penile SCCs than in healthy penile tissues [8]. Since then, additional studies have examined asymptomatic horses [15, 16, 21].

Our research group has recently detected EcPV-2 in penile SCCs and papillomas from Brazilian horses [17], with EcPV-2 DNA identified in approximately 18% of the examined penile tumors. Previous studies have reported variable but generally higher prevalence rates. EcPV-2 has been detected in 29% of genital tumors in Canadian horses [19], 43–45% of penile tumors in North American horses [8, 9], and up to 100% of penile tumors in Swiss horses [12].

Studies investigating the presence of EcPV-2 in healthy equine genital mucosa have produced variable results. While some studies report relatively high detection rates in asymptomatic horses (29–30%) [15, 16], others have identified the virus in only a small proportion of animals (1–6.7%) [10, 11, 13, 21]. In the present study, EcPV-2 DNA was detected in one horse (1.9%) within the investigated population. Greenwood et al. [15] conducted one of the most comprehensive investigations of EcPV-2 in asymptomatic horses, identifying virus DNA in 29% (20/70) of tissue samples collected at necropsy. In the same study, a parallel serological survey in live asymptomatic horses revealed antibodies against EcPV-2 in 36% of animals. Differences among studies may be related to methodological variations, including sampling techniques and PCR protocols. For example, while Sykora et al. [10], Bogaert et al. [11], and Lee et al. [21] used cotton swabs for mucosal sampling, Capelli et al. [16] and Fischer et al. [14] used mucosal cytobrushes and Greenwood et al. [15] analyzed tissue fragments collected at necropsy. Cotton swabs appear less effective for papillomavirus molecular detection compared with cytobrushes [22], likely due to the virus’s tropism for basal epithelial layers [4], which may limit the sensitivity of superficial mucosal sampling. Although our study adopted a necropsy-based sampling method similar to that used by Greenwood et al. [15], the number and size of tissue fragments analyzed for molecular testing were not specified in that study, which complicates direct comparisons. In addition, differences in PCR methodology, including the use of quantitative PCR, may also influence detection rates [13, 16]. Indeed, one of the few studies employing this approach detected EcPV-2 DNA in only 2.3% of samples from healthy genital mucosa [13]. The inclusion of mares in our sample set may also have influenced the detection frequency, as EcPV-2 has historically been more commonly associated with penile than vulvar lesions [13], although recent studies have reported relatively high detection rates in asymptomatic mares [15, 16]. In addition, the inclusion of very young animals in the study population, including aborted fetuses, may also have influenced the detection rate, since EcPV-2 infection is most frequently reported in sexually mature horses [15, 19]. However, EcPV-2 DNA has previously been detected by PCR in aborted equine fetuses, suggesting the possibility of vertical transmission and supporting the inclusion of these specimens in investigations of viral occurrence [19]. An additional limitation of the present study that may have contributed to the low frequency of detection relates to DNA quality control during PCR amplification. The inclusion of a housekeeping gene (GAPDH) could have provided additional assurance of DNA integrity in the analyzed samples. Although such controls were not included in the present assay, the unequivocal amplification and sequencing of EcPV-2 DNA in one sample nevertheless confirms the presence of the virus in a clinically normal horse from southern Brazil. In addition, the molecular approach employed in this study was limited to conventional PCR for viral DNA detection. Consequently, the results do not allow conclusions regarding viral activity, viral load, or transcriptional status. Analyses such as quantitative PCR or the evaluation of viral oncogene expression (e.g., E6/E7 transcripts) would be necessary to determine whether the detected viral DNA reflects active infection, viral persistence, or transient viral presence. Moreover, because DNA extraction was performed from pooled tissues, it was not possible to determine the specific anatomical site harboring viral DNA. Despite these limitations, the detection and sequence confirmation of EcPV-2 DNA in a clinically normal horse provides evidence that the virus can occur in asymptomatic animals and supports the need for further studies to better understand the epidemiology and biological significance of EcPV-2 infection in equine populations.

Although not yet fully elucidated, EcPV-2 transmission is thought to occur through direct contact between animals (including sexual transmission), via contaminated fomites, and possibly vertically [15, 23, 24]. Subclinical PV infection appears to be influenced by a combination of host, viral, and environmental factors [8, 25], although age and sex may not be as significant for EcPV-2 infection as previously assumed [15, 16]. In the present study, EcPV-2 DNA was detected in one clinically normal horse. Although the frequency of detection was low, this finding indicated that the virus can be present in asymptomatic animals and is consistent with previous reports suggesting that clinically normal horses may harbor EcPV-2 [15, 16]. Such animals could potentially contribute to viral maintenance and transmission within equine populations. PV infection does not necessarily lead immediately to tumorigenesis in mammals. In humans, PV-induced carcinogenesis may take more than a decade to develop, during which infected individuals may remain asymptomatic carriers capable of viral transmission [26]. Moreover, subclinical human PV infection in humans is more frequent than clinical disease, with many infections being spontaneously cleared [26, 27]. The host immune response plays an important role during this period, as many individuals eliminate the virus before persistent infection becomes established [27], whereas others may fail to clear the infection and subsequently progress to carcinogenesis [16, 27]. In this context, the detection of EcPV-2 DNA in an asymptomatic horse could represent either a transient infection that may eventually be cleared or a persistent infection with potential long-term implications, although further studies are required to better understand the natural history of EcPV-2 infection in horses [16].

## Conclusion

This study provides the first evidence of EcPV-2 DNA in the genital mucosa of an asymptomatic horse in Brazil, indicating that clinically normal animals may harbor the virus in the local equine population. Although the frequency of detection was low and may have been influenced by factors such as DNA quality and other methodological aspects discussed above, these findings expand the current understanding of EcPV-2 beyond clinically affected horses.

## Supplementary Information

Below is the link to the electronic supplementary material.

Supplementary Material 1 (DOCX 30.4 KB)

Supplementary Material 2 (JPG 22.7 KB) Supplementary Fig. 1 Phylogenetic tree of equine papillomavirus sequences. The tree was constructed from a 156 bp fragment obtained from the PCR product sequenced in this study. Sequences were aligned using MUSCLE in Geneious and the tree was inferred using the neighbor-joining method. Reference sequences of Equine papillomavirus type 2 and Equine papillomavirus type 1 (NC_003748.1) were retrieved from GenBank, with EcPV-1 included as an outgroup. Branch support values are shown at the nodes (as proportions), and the scale bar indicates the number of nucleotide substitutions per site. The sequence obtained in this study (Sample_study 2 consensus sequence) clustered with EcPV-2.
