# Supplementary material for: Search for Equus caballus papillomavirus type 2 in tissues of asymptomatic horses in southern brazil
Source: Braz J Microbiol. 2026 Apr 22;57(1):113. doi: 10.1007/s42770-026-01930-y (PMC13103048; doi:10.1007/s42770-026-01930-y)
Supplement: Supplementary file 1 — Supplementary Material 1 (DOCX 30.4 KB) [file 42770_2026_1930_MOESM1_ESM.docx]

| **N** | **sex** | **Breed** | **Age** | **Municipality** |  | **Collected samples** | | | | | **EcPV-2 amplification** |
| --- | --- | --- | --- | --- | --- | --- | --- | --- | --- | --- | --- |
|  |  |  |  |  | **Sample source** | **Penile or vulvar mucosa** | **Anal mucosa** | **Lip** | **Lower eyelid** | **Stomach** |  |
| 1 | M | Criollo | 2 y | n.i. | N | x | x | x | x | x | - |
| 2 | M | Mixed Breed | 10 y | Santa Maria | N | x | x | x | x | x | - |
| 3 | F | Criollo | 40 y | Nova Palma | N | x | x | x | x | x | - |
| 4 | M | Lusitano | 7 y | Jaguari | N | x | x | x | x | x | - |
| 5 | F | Thoroughbred | 10 y | Santa Maria | N | x | x | x | x | x | - |
| 6 | M | Mixed Breed | 20 y | Santa Maria | N | x | x | x | x | x | - |
| 7 | M | Criollo | 9 y | Santa Maria | N | x | x | x | x | x | - |
| 8 | M | Brazilian Sport Horse | 25 y | Santa Maria | N | x | x | x | x | x | - |
| 9 | F | Criollo | 5 y | Uruguaiana | N | x | x | x | x | x | - |
| 10 | F | Thoroughbred | 13 y | Aceguá | N | x | x | x | x | x | - |
| 11 | F | Criollo | 1 d | Santa Maria | N | x | x | x | x | x | - |
| 12 | M | Mixed Breed | 8 y | n.i. | N | x | x | x | x | x | - |
| 13 | F | Crioulo | 6 y | Uruguaiana | N | x | x | x | x | x | - |
| 14 | F | Crioulo | 21 y | Santa Maria | N | x | x | x | x | x | - |
| 15 | F | Crioulo | 10 y | n.i. | N | x | x | x | x | x | - |
| 16 | F | Crioulo | 35 d | Santa Maria | N | x | x | x | x | x | - |
| 17 | F | Quarter Horse | 16 y | Santa Maria | N | x | x | x | x | x | - |
| 18 | M | Thoroughbred | 12 y | Bagé | N | x | x | x | x | x | - |
| 19 | M | Criollo | 10 y | São Gabriel | N | x | x | x | x | x | **+** |
| 20 | M | Thoroughbred | 50 d | Bagé | N | x | x | x | x | x | - |
| 21 | M | Criollo | 14 d | Uruguaiana | N | x | x | x | x | x | - |
| 22 | F | Criollo | 30 d | Santa Maria | N | x | x | x | x | x | - |
| 23 | F | Brazilian Sport Horse | n.i. | n.i. | N | x | x | x | x | x | - |
| 24 | F | Thoroughbred | 60 d | Bagé | N | x | x | x | x | x | - |
| 25 | M | Thoroughbred | 30 d | Bagé | N | x | x | x | x | x | - |
| 26 | M | Thoroughbred | 60 d | Bagé | N | x | x | x | x | x | - |
| 27 | F | Thoroughbred | 31 d | n.i. | N | x | x | x | x | x | - |
| 28 | M | Brazilian Sport Horse | 11 d | Santa Maria | N | x | x | x | x | x | - |
| 29 | M | Thoroughbred | 50 d | Bagé | N | x | x | x | x | x | - |
| 30 | M | Mixed Breed | 21 y | Santa Maria | N | x | x | x | x | x | - |
| 31 | M | Thoroughbred | 29 d | Aceguá | N | x | x | x | x | x | - |
| 32 | F | Thoroughbred | 10 y | n.i. | N | x | x | x | x | x | - |
| 33 | F | Criollo | 7 y | Alegrete | N | x | x | x | x | x | - |
| 34 | F | Thoroughbred | 3 y | Bagé | N | x | x | x | x | x | - |
| 35 | M | Criollo | 2 y | Santa Maria | N | x | x | x | x | x | - |
| 36 | F | Thoroughbred | 4 y | Bagé | N | x | x | x | x | x | - |
| 37 | M | Criollo | 25 y | Santa Maria | N | x | x | x | x | x | - |
| 38 | M | Thoroughbred | 27 y | Santa Maria | N | x | x | x | x | x | - |
| 39 | F | Thoroughbred | 14 y | Bagé | N | x | x | x | x | x | - |
| 40 | M | Thoroughbred | 2 y | Bagé | N | x | x | x | x | x | - |
| 41 | M | n.i. | n.i. | n.i. | A | x | - | - | - | - | - |
| 42 | M | n.i. | n.i. | n.i. | A | x | - | - | - | - | - |
| 43 | M | n.i. | n.i. | n.i. | A | x | - | - | - | - | - |
| 44 | M | n.i. | n.i. | n.i. | A | x | - | - | - | - | - |
| 45 | M | n.i. | n.i. | n.i. | A | x | - | - | - | - | - |
| 46 | M | n.i. | n.i. | n.i. | A | x | - | - | - | - | - |
| 47 | M | n.i. | n.i. | n.i. | A | x | - | - | - | - | - |
| 48 | M | n.i. | n.i. | n.i. | A | x | - | - | - | - | - |
| 49 | M | n.i. | n.i. | n.i. | A | x | - | - | - | - | - |
| 50 | M | n.i. | n.i. | n.i. | A | x | - | - | - | - | - |
| 51 | M | n.i. | n.i. | n.i. | A | x | - | - | - | - | - |
| 52 | M | n.i. | n.i. | n.i. | A | x | - | - | - | - | - |
| 53 | M | n.i. | n.i. | n.i. | A | x | - | - | - | - | - |
| 54 | M | n.i. | n.i. | n.i. | A | x | - | - | - | - | - |

**Supplementary Table 1. Age, sex, breed, municipality of origin, sample source, collected tissues, and EcPV-2 PCR results for each horse included in the study**

y: years; m: months; d: days; n.i.: not informed; N: necropsy; A: abattoir
