# Supplementary figures and images for: Search for Equus caballus papillomavirus type 2 in tissues of asymptomatic horses in southern brazil
Source: Braz J Microbiol. 2026 Apr 22;57(1):113. doi: 10.1007/s42770-026-01930-y (PMC13103048; doi:10.1007/s42770-026-01930-y)

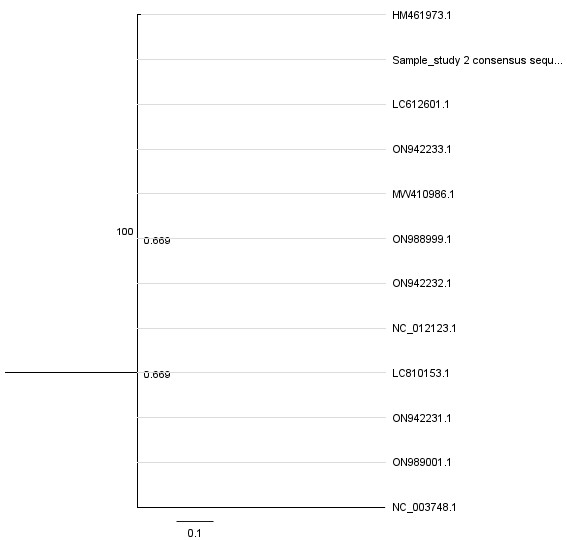

Supplement: Supplementary file 2 — Supplementary Material 2 (JPG 22.7 KB) Supplementary Fig. 1 Phylogenetic tree of equine papillomavirus sequences. The tree was constructed from a 156 bp fragment obtained from the PCR product sequenced in this study. Sequences were aligned using MUSCLE in Geneious and the tree was inferred using the neighbor-joining method. Reference sequences of Equine papillomavirus type 2 and Equine papillomavirus type 1 (NC_003748.1) were retrieved from GenBank, with EcPV-1 included as an outgroup. Branch support values are shown at the nodes (as proportions), and the scale bar indicates the number of nucleotide substitutions per site. The sequence obtained in this study (Sample_study 2 consensus sequence) clustered with EcPV-2. [file 42770_2026_1930_MOESM2_ESM.jpg]
